# Parental Longevity and Cognitive Ability among Older Adults in Mexico

**DOI:** 10.1002/alz70860_100559

**Published:** 2025-12-23

**Authors:** Joseph Saenz, Laura Tanner

**Affiliations:** ^1^ Arizona State University, Phoenix, AZ, USA; ^2^ Arizona State Univeristy, Phoenix, AZ, USA

## Abstract

**Background:**

Prior studies find better cognitive outcomes in offspring of parents with extended longevity. Few evaluate how parental longevity relates with cognitive ability in low‐ and middle‐income countries, or the potential economic and health mechanisms that may explain these associations. This study evaluates associations between parental longevity and offspring cognitive ability, and the underlying mechanisms, within Mexico, an upper middle‐income country.

**Method:**

We used data from the nationally representative 2018 Mexican Health and Aging Study (*n* = 7,295, age 60+). MHAS study participants self‐reported longevity of each of their parents, which we categorized based on whether each parent passed away before age 80 versus 80+. We used linear regression to estimate associations between parental longevity, general cognitive ability. and cognitive tasks spanning multiple cognitive domains. adjusting for confounders and potential economic (offspring education), health (chronic condition count), and health behavior (smoking, binge drinking) mechanisms.

**Result:**

In the MHAS sample, 45.4% of participants reported their mother lived to age 80 or beyond and 42.6% reported their father lived to age 80 or beyond. In models adjusting for age, sex, parental education, and early‐life SES, maternal longevity related with higher general cognitive ability and all cognitive tasks (Verbal Learning, Verbal Recall, Visual Scanning, Verbal Fluency, Visuospatial Ability, and Visual Recall). Paternal longevity was unrelated with cognition. Associations between maternal longevity and cognition were reduced with further adjustment for education but remained statistically significant. Adjustment for health and health behaviors did not meaningfully impact associations between maternal longevity and cognition.

**Conclusion:**

Our results suggest that maternal, though not paternal, longevity is positively related with cognition in their offspring. The benefits of maternal longevity could be partially attributed to higher education in offspring of long‐lived mothers. Other factors such as genetics and familial support may also explain higher cognitive ability in offspring of long‐lived mothers.

